# Estrogen-like activity of seafood related to environmental chemical contaminants

**DOI:** 10.1186/1476-069X-5-9

**Published:** 2006-03-30

**Authors:** Sonia Garritano, Barbara Pinto, Marco Calderisi, Teresa Cirillo, Renata Amodio-Cocchieri, Daniela Reali

**Affiliations:** 1 IARC, 150 Cours Albert-Thomas, 69372 Lyon Cedex 08, France; 2 Department of Experimental Pathology, Medical Biotechnology, Infectivology and Epidemiology, University of Pisa, Via San Zeno 37, 56127 Pisa, Italy; 3 Department of Food Science, University of Naples Federico II, Via Università 100, 80055 Portici, Italy

## Abstract

**Background:**

A wide variety of environmental pollutants occur in surface waters, including estuarine and marine waters. Many of these contaminants are recognised as endocrine disrupting chemicals (EDCs) which can adversely affect the male and female reproductive system by binding the estrogen receptor and exhibiting hormone-like activities. In this study the estrogenic activity of extracts of edible marine organisms for human consumption from the Mediterranean Sea was assayed.

**Methods:**

Marine organisms were collected in two different areas of the Mediterranean Sea. The estrogenic activity of tissues was assessed using an *in vitro *yeast reporter gene assay (*S. cerevisiae *RMY 326 ER-ERE). Concentrations of polychlorinated biphenyls (PCBs) (congeners 28, 52, 101, 118, 138, 153, 180) in fish tissue was also evaluated.

**Results:**

Thirty-eight percent of extracts showed a hormone-like activity higher than 10% of the activity elicited by 10 nM 17b-estradiol (E2) used as control.

Total PCB concentrations ranged from 0.002 up to 1.785 ng/g wet weight. Chemical analyses detected different levels of contamination among the species collected in the two areas, with the ones collected in the Adriatic Sea showing concentrations significantly higher than those collected in the Tyrrhenian Sea (p < 0.01).

**Conclusion:**

The more frequent combination of chemicals in the samples that showed higher estrogenic activity was PCB 28, PCB 101, PCB 153, PCB 180.

The content of PCBs and estrogenic activity did not reveal any significant correlation.

## Background

A wide variety of environmental pollutants occur in surface waters, including estuarine and marine waters. Many of these contaminants are recognised as endocrine disrupting chemicals (EDCs) and include substances of very different chemical property such as therapeutic agents, alkylphenols, dioxins, pesticides, plasticizers and surfactants. Of particular concern are the polychlorinated biphenyls (PCBs) because of their ubiquity and their lipophilic and persistent nature (they tend to accumulate within the fat and tissue of animals and humans). These compounds are widely spread in the environment despite most countries have banned their production. They continue to be detected in the ecosystem including marine habitat because of deliberate or accidental dumping or through disposal of goods containing them. There is evidence that these chemicals can adversely affect the male and female reproductive system by binding the estrogen receptor and exhibiting estrogenic or anti-estrogenic activities [1-3]. The susceptibility of target tissues is related to the stage of development, the immune status of the individual and the cumulative exposure dose. Twelve PCBs congeners, PCBs 77, 81, 126 and 169 (non-*ortho *PCBs), PCBs 105, 114, 118, 123, 156, 157, 167 and 189 (*ortho *PCBs), were identified by the WHO as having dioxin-like properties, and 7 congeners (PCBs 28, 52, 101, 118, 153, 138, 180) were identified by the International Council for the Exploration of the Seas (ICES) as markers of the degree of contamination. These compounds are the second greatest cause of fish advisory, according to US-EPA [4]. The main source of exposure to PCBs for humans is represented by food, specifically of animal origin [5-7]. In epidemiological studies, PCBs have been associated with immunotoxicity [8] and neurobehavioral deficits have been reported in children prenatally exposed to PCBs and through mother's milk [9] or ascribed to the consumption of PCB contaminated food including fish accumulating these substances directly from the surrounding environment [10-12].

In this study the estrogenic activity of extracts of edible marine organisms from two areas of the Mediterranean Sea was assayed in order to evaluate the exposure of edible species of the Mediterranean Sea largely consumed in the Italian diet to complex mixtures of xenobiotics that may exhibit estrogenic activities. We also evaluated the concentration of ICES-7 PCBs considered as target compounds in marine pollution studies.

## Methods

### Sampling

Fish, crustaceans and cephalopods were collected directly from professional fishing in two areas of the Mediterranean Sea, the Adriatic Sea within 40 miles S-E from the Pescara port, and Tyrrhenian Sea within 50 miles S-W from the Naples port, respectively, from June to July 2004. The organisms collected in the Adriatic Sea were Blue-mouth (*Helicolenus dactylopterus*), Broad-tail shortfin squid (*Illex coindetii*), Red mullet (*Mullus barbatus*), European hake (*Merluccius merluccius*), Fork beard (*Phycis phycis*), Deepwater rose shrimp (*Parapenaeus longirostris*), Atlantic mackerel (*Scomber scombrus*); in Tyrrhenian Sea were Common squid (*Loligo vulgaris*), Red mullet (*Mullus barbatus*), Common grey mullet (*Mugil cephalus*), Fork beard (*Phycis phycis*), Blue whiting (*Micromesistius poutassou*), Common octopus (*Octopus vulgaris*), Gilt-head seabream (*Sparus aurata*), Common cuttlefish (*Sepia officinalis*), Atlantic mackerel (*Scomber scombrus*). These organisms, that represent different trophic positions in the marine environment, were selected because they are abundant, widely distributed in the Mediterranean area and available all over the year and also because they represent a largely consumed seafood. The specimens, all of commercial size, were wrapped in aluminium foil, then immediately refrigerated and transported to the laboratory.

### Analytical sample preparation

Each species was classified; the length and weight of each specimen were measured and recorded. The specimens with weight > 200 g were individually analysed, those with weights < 200 g were pooled, obtaining 20 analytical samples from the Adriatic Sea and 22 from the Tyrrhenian Sea. The edible part of the marine organisms was selected, homogenised, and subsequently lyophilised.

### Fat extraction

Fat was cold-extracted from lyophilised tissues with petroleum ether/acetone (1:1, v/v). The extract was passed through a glass tube packed with anhydrous sodium sulphate and then evaporated by rotavapor (40°C and low pressure) and the lipid residue was weighed.

### Chemical analysis

The cleanup of fat extracts (50 mg) was carried out on Extrelut-NT3/Extrelut-NT1 cartridges (Merck KGaA – Darmstadt, Germany) with the addition of 0.36 g of C-18 Isolute (40–60 mesh Merck KGaA Darmstad, Germany) and eluted with acetonitrile. The extracts were concentrated under vacuum at 40°C, cleaned up by column adsorption-chromatography on Florisil (60/100 mesh – Supelco Bellefonte, PA USA) activated at 130°C for 2 h and eluted with 30 ml n-hexane added in 5 ml aliquots. The eluate was concentrated to a small volume (< 1 ml) by evaporation at room temperature under a flow of N_2_, and 1 ml isooctane was added as a keeper. Seven PCB congeners (IUPAC nn. 28, 52, 101, 118, 153, 138 and 180) were detected in seafood according to the analytical method of Italian Public Health laboratories [13].

For PCBs quantification, samples were injected into a capillary gas chromatographer with electron capture detector (GC-ECD) with Temperature program 60°C for 2 minutes, increasing of 10°C/min to 170°C stay for 2 minutes, increasing of 2°C/min to 210°C, increasing for 10°C/min to 260°C. GC-MS was used for their confirm. The internal standard solution (PCB 209) was added to the extract before injecting. The evaluation of PCB concentrations in the samples was carried out by comparison with a calibration curve obtained by a pool of the seven ICES PCB congeners: PCB 28 (2, 4, 4' tri-chlorobiphenyls), PCB 52 (2, 2', 5, 5' tetra-chlorobiphenyls), PCB 101 (2, 4, 5, 2', 5' penta-chlorobiphenyls), PCB 118 (2, 4, 5, 3', 4' penta-chlorobiphenyls), PCB 138 (2, 2'3, 4, 4', 5' hexa-chlorobiphenyls) PCB 153 (2, 2', 4.4', 5, 5' hexa-chlorobiphenyls) and PCB 180 (2, 2', 3, 4, 4', 5, 5' hepta-chlorobiphenyls). All of the compounds (95% – 99% pure) were purchased from Dr. Ehrenstorfer (GmbH, Augsburg, Germany). In the analytical conditions applied, the detection limits were: 0.002 ng/g wet weight (w wt) for the PCBs nn 180 and 138; 0.003 ng/g (w wt) for the PCB n 153; 0.005 ng/g (w wt) for the PCBs nn 52, 101, 118 and 0.008 ng/g (w wt) for the PCB n 28. The mean recovery obtained by PCB standard spiked samples was 70 ± 7%. Total PCB levels were calculated as the sum (∑) of all the seven determined congeners.

### Yeast strain

Estrogenic activity of organic extracts of seafood (200 g wet weight) and standard PCBs was tested by *S. cerevisiae *yeast strain (RMY326 ER-ERE) containing the human estrogen receptor (hERa) and a *Xenopus laevis *vitellogenin estrogen-responsive element (ERE) linked to a reporter gene lac *Z *encoding for the enzyme β-galactosidase. Plasmid pG/ER(G) was used as the yeast expression vector for ERα and PUCΔSS-ERE as its b-galactosidase reporter plasmid [14,15]. The sensitivity and specificity of the yeast strain were previously assessed using 17b-estradiol (E2), diethylstilbestrol (DES), and other natural and synthetic chemicals [16,17].

### Medium

A synthetic drop-out selective medium lacking uracil and thryptophane to maintain plasmid selection was prepared by adding 0.67 g yeast nitrogen base, 2% glucose, 10 ml a stock aminoacids solution (30 mg L-isoleucine, 150 mg L-valine, 20 mg L- arginine-HCl, 30 mg L-lysine-HCl, 20 mg L-methionine, 50 mg L-phenylalanine, 200 mg L-threonine, 30 mg L-tyrosine in 100 ml water), 1 ml a stock L-hystidine-HCl solution (200 mg in 100 ml water), 1 ml a stock L- leucine solution (1 gr in 100 ml water) and an adenine hemisulfate solution (200 mg in 100 ml water) to 90 ml water endotoxin-free cell cultures (Sigma). Bacto-Agar (DIFCO) (3 g) was used for the solid media.

### Yeast assay

Yeast cultures were incubated at 28°C for 7 hr by continuously shaking on an orbital shaker (210 rpm) in 1 ml of selective medium. The cultures were then diluted in fresh medium to an optical density of 0.1 (OD_600 _nm) and incubated at 30°C for 17 h (overnight) in the absence or presence of 17b-estradiol (positive control), solvent (negative control), pure chemicals and organic extracts. Dimethylsulphoxide (DMSO) was used as solvent. Solutions of standard PCB congeners and extracts were evaporated under a gentle flow of nitrogen and the pellet was resuspended in 10 μl DMSO.

PCB standards were tested at 5 μg/ml. Congeners 138 and 153 were assayed at 0.05 μg/ml because at higher concentrations strongly inhibited yeast cells growth. The samples were added to the yeast culture so that the concentration of solvent DMSO did not exceed 1% (v/v).

### b-galactosidase assay

Yeast cells were harvested by centrifugation and the pellet was resuspended in 1 ml of Z-buffer (60 mM Na_2_HPO_4_·7H_2_O, 40 mM NaH_2_HPO_4_·H_2_O, 10 mM KCl, 1 mM MgSO_4_·7H_2_O, and 35 mM 2-mercaptoethanol, pH 7.0). After centrifugation, the pellet was resuspended in 150 μl of Z-buffer. The cells were permeabilized by adding 50 μl dichloromethane, 20 μl 0.1% SDS, 5–50 μl resuspended cells (out of 150 μl) plus Z-buffer for a total of 150 μl including cells, followed by vortexing for 10 s. The enzymatic reaction was started by adding 700 μl 2 mg/ml o-nitrophenyl b-D-galactopyranoside (ONPG) to the Z-buffer and incubating at 30°C for 5–10 min. The reaction was terminated by the addition of 500 μl 1 M Na_2_CO_3 _and the absorbance at 420 nm (OD_420_) of the sample was measured. The b-gal activity was normalised to the number of cells assayed and expressed as Miller units using the following formula [18]:

b-gal units (M.U.) = (1000 × OD_420_)/(t × V × OD_600_)

t = length of incubation (min) V = volume of culture used in the assay (ml)

**Figure 1:**
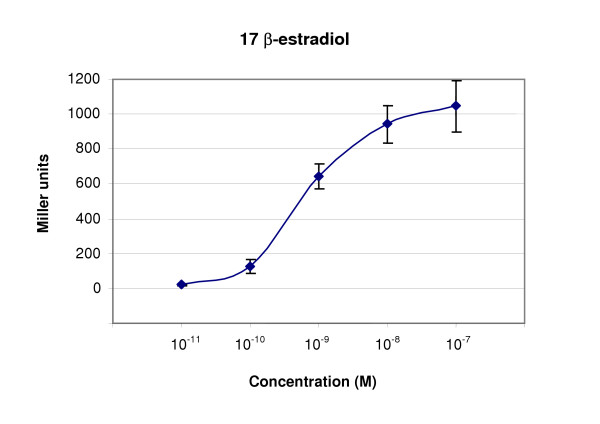
**Dose-response curve of E2 concentrations**. Data represent the mean ± S.D. of sixteen independent experiments.

The b-galactosidase activity induction elicited by estradiol, the reference estrogen, showed a sigmoid shape (Figure 1) and adequately fitted a linear dose-response relationship after Log transformation of Miller Units.

The b-galactosidase activity of the samples was expressed as a percentage of the activity obtained with 10 nM E2 (positive control) [19].

### Statistical analysis

Chemo-analytical and biological results were processed using Principal Components Analysis (PCA) [20,21] and Partial Least Square regression (PLS) [22]. Principal Components Analysis is a rotation of the original data in order to orientate the first new axis in the direction of the maximum explained variance. The second new axis will be oriented perpendicularly to the first, in order to maximise the residual variance, and so on until all the information of the system is explained. Using Principal Components Analysis is possible to:

a) estimate correlation between variables

b) display objects (finding outliers, clusters, ...)

c) summarise the "information" of a system

d) reduce dataset dimension

e) find mean aspect of the system

PLS regression is a biased regression method that allow to obtain very stable models and it can be used also when the ratio cases/predictors is smaller then one and when there are predictors that are strongly correlated.

This method uses predictors PCs and responses to find the couple that as got the best correlation and goes on using other PCs until there is still usable "information". At the end the number of PLS components to be used is the one that let maximise the explained variance in prediction.

Expected estrogenicity according to the content of individual PCBs measured in each sample was calculated from the chemical data for each sample by using the principle of concentration additivity and relative potencies of the various chemicals as determined with the yeast estrogen screen.

The use of Log transformation of PCB and b-galactosidase activity values normalised their distribution and stabilised the variance allowing the use of parametric methods. The Statgraphics Plus statistical package was used (Magnugistic, Rockville MD, USA).

## Results

### PCBs assessment

**Figure 2:**
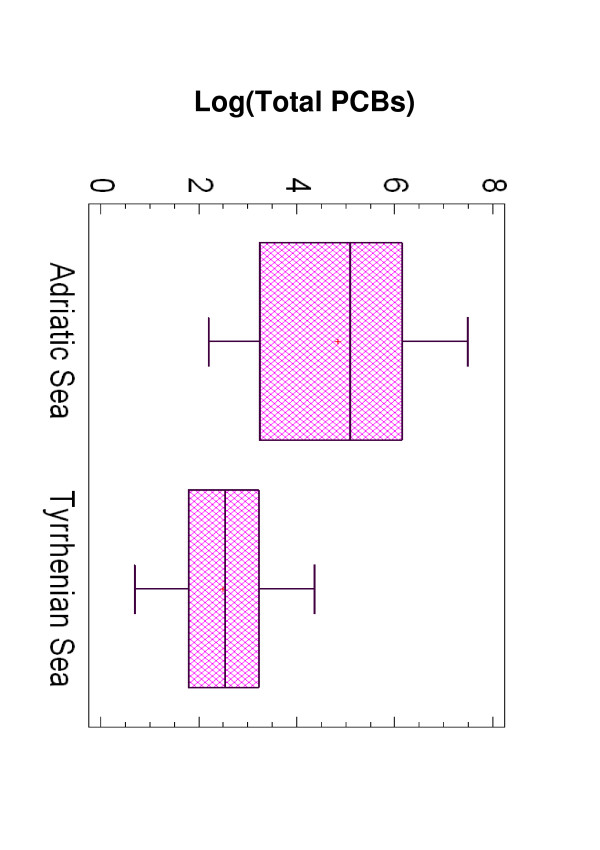
Differences in total PCBs content (Log) in samples from the Adriatic and the Tyrrhenian Sea.

Twenty samples of marine organisms from the Adriatic Sea and 22 from the Tyrrhenian Sea, mainly belonging to species, *M. barbatus*, *S. scombrus*, *P. phycis*, *M. merluccius *and *O. vulgaris *were analysed for the presence of seven reference PCBs, namely PCB 28, PCB 52, PBC 101, PCB 118, PCB 138, PCB 153 and PCB 180. They were chosen because considered to be suitable indicators of industrial marine pollution by International Agencies. Forty-one percent of 294 chemical determinations gave negative results (under the detection limit). Total PCB concentrations ranged from 0.002 up to 1.785 ng/g wet weight. Species from the Adriatic Sea were more contaminated than those collected in the Tyrrhenian Sea (Table 1) and the analysis of variance showed that this difference was statistically significant (*p *= 0.002) (Figure 2).

**Table 1:** PCB concentrations (ng/g wet weight) determined in marine species collected in Adriatic and Tyrrhenian Sea

**Adriatic Sea samples**	**Fat (g%)**	**PCB 28**	**PCB 52**	**PCB 101**	**PCB 118**	**PCB 138**	**PCB 153**	**PCB 180**	**PCB**
*1. H. dactilopterus*	0.3	ND^a^	ND	ND	ND	73	8	n.d	81
*2. I. coindetii*	0.5	ND	ND	ND	ND	37	42	109	188
*3. I. coindetii*	0.5	ND	ND	ND	ND	26	41	72	139
*4. M. barbatus*	1.7	ND	ND	ND	76	184	264	208	732
*5. M. barbatus*	1.4	ND	ND	15	ND	123	168	137	443
*6. M. merluccius*	0.4	46	4	7	10	21	27	11	126
*7. M. merluccius*	0.8	41	4	10	12	37	48	52	204
*8. M. merluccius*	0.9	44	5	18	23	69	82	29	270
*9. M. merluccius*	0.4	ND	39	14	ND	56	66	21	196
*10. M. merluccius*	0.4	ND	ND	ND	ND	ND	9	ND	9
*11. P. phycis*	0.6	15	ND	ND	55	ND	ND	ND	70
*12. P. phycis*	0.6	ND	ND	ND	ND	6	7	12	25
*13. P. phycis*	0.8	ND	1	ND	10	ND	6	ND	17
*14. P. phycis*	0.5	ND	1	ND	8	ND	5	ND	14
*15. P. phycis*	0.7	ND	ND	ND	ND	6	8	12	26
*16. P. longirostris*	0.4	ND	2	ND	ND	2	4	8	16
*17. S. scombrus*	0.5	244	ND	135	ND	497	196	92	1164
*18. S. scombrus*	0.6	ND	ND	ND	ND	ND	78	400	478
*19. S. scombrus*	0.6	1469	191	ND	ND	ND	ND	125	1785
*20. S. scombrus*	0.7	ND	ND	ND	ND	320	311	6	637

**Tyrrhenian Sea samples**	**Fat (g%)**	**PCB 28**	**PCB 52**	**PCB 101**	**PCB 118**	**PCB 138**	**PCB 153**	**PCB 180**	**PCB**

									
*1. L. vulgaris*	0.7	12	ND^*a*^	ND	ND	ND	ND	ND	12
*2. L. vulgaris*	0.6	18	ND	ND	ND	1	ND	ND	19
*3. M. barbatus*	1.3	5	ND	ND	1	ND	5	2	13
*4. M. barbatus*	1.2	ND	ND	ND	ND	ND	1	1	2
*5. M. barbatus*	1.4	10	ND	15	ND	ND	ND	1	26
*6. M. cephalus*	0.6	7	1	1	ND	ND	ND	ND	9
*7. P. phycis*	0.5	6	ND	1	3	ND	6	2	18
*8. P. phycis*	0.7	17	6	ND	ND	ND	2	ND	25
*9. P. phycis*	1.5	ND	ND	1	ND	ND	2	4	7
*10. P. phycis*	0.8	3	ND	ND	ND	ND	3	ND	6
*11. M. poutassou*	0.4	ND	ND	13	9	1	2	1	26
*12. M. poutassou*	0.4	8	3	27	25	15	ND	ND	78
*13. O. vulgaris*	0.6	2	ND	1	ND	ND	ND	ND	3
*14. O. vulgaris*	0.7	5	ND	ND	ND	ND	ND	ND	5
*15. O. vulgaris*	0.6	17	ND	2	3	ND	1	1	24
*16. O. vulgaris*	0.5	5	ND	ND	ND	ND	ND	ND	5
*17. S. aurata*	1.2	1	2	11	16	ND	ND	ND	30
*18. S. officinalis*	0.6	ND	ND	ND	ND	ND	ND	3	3
*19. S. officinalis*	0.6	4	ND	ND	ND	ND	3	2	9
*20. S. scombrus*	0.4	4	ND	3	ND	3	ND	ND	10
*21. S. scombrus*	0.3	7	ND	3	10	ND	3	2	25
*22. S. scombrus*	0.8	5	ND	16	ND	4	6	3	34

^a^ND detection limit

**Figure 3:**
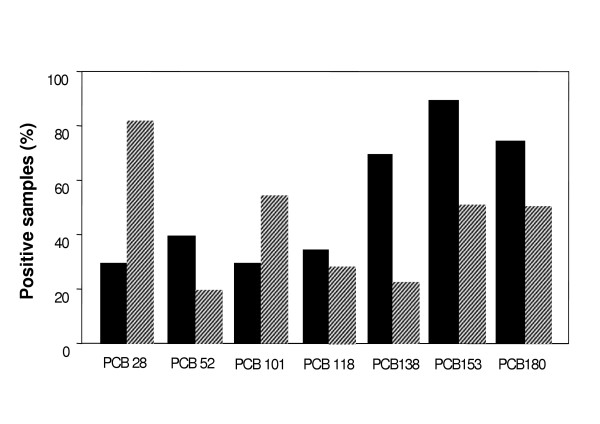
**Distribution of individual PCB congeners in samples from the Adriatic Sea (■) and the Tyrrhenian (▨) Sea**. Species from the Adriatic Sea were more contaminated than those collected in the Tyrrhenian Sea. Number of positive samples (n) for individual PCB congener in the Adriatic Sea: PCB 28 (n = 6), PCB 52 (n = 8), PCB 101 (n = 6), PCB 118 (n = 7), PCB 138 (n = 14), PCB 153 (n = 18), PCB 180 (n = 15). Number of positive samples (n) for individual PCB congener in the Tyrrhenian Sea: PCB 28 (n = 18), PCB 52 (n = 4), PCB 101 (n = 12), PCB 118 (n = 7), PCB 138 (n = 5), PCB 153 (n = 11), PCB 180 (n = 11).

Individual PCB congeners distribution also differed depending on the area of fishing (Figure 3). The species collected in the Adriatic Sea were more often contaminated with hexa- and heptachlorobiphenyls, and this is in accord to studies from other authors [23], whereas samples collected in the Tyrrhenian Sea were mostly contaminated by PCB 28.

**Figure 4:**
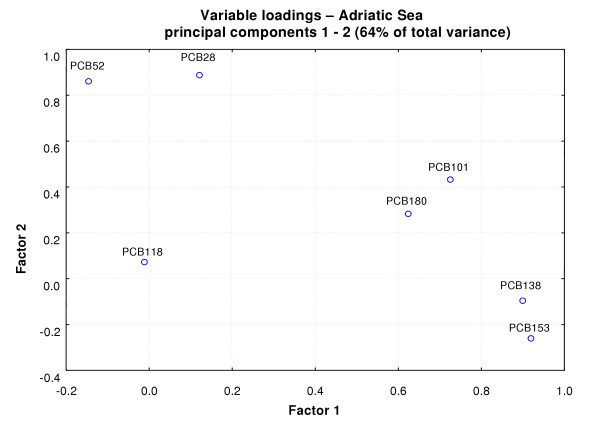
Principal components analysis loading plot of PCBs contamination in samples collected in the Adriatic Sea.

**Figure 5:**
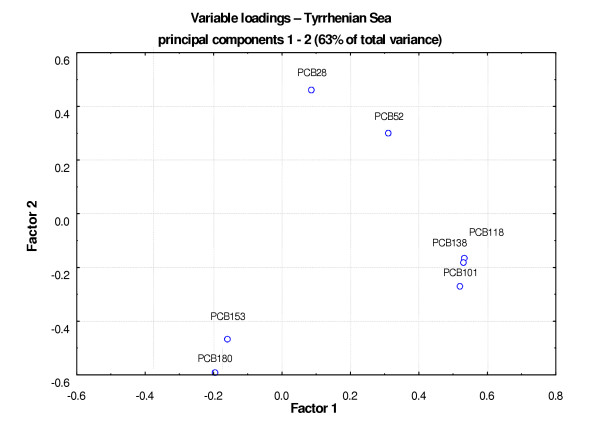
Principal components analysis loading plot of PCBs contamination in samples collected in the Tyrrhenian Sea.

Multivariate statistical analysis (PCA) showed different groups of correlation between the PCBs in the two datasets. The first two principal components are able to explain 64% of variance in the Adriatic dataset, and 63% of variance in the Thyrrenian dataset (Figures 4, 5).

Taking into account the species, the multifactor analysis of variance showed that the difference in PCBs contamination observed in the two seas is mainly due to the PCBs content of *S. scombrus *and *M. barbatus*. These species showed for samples collected in the Adriatic Sea, higher concentrations of total PCBs (Table 1), accordingly to other authors [24].

### b-galactosidase activity

**Figure 6:**
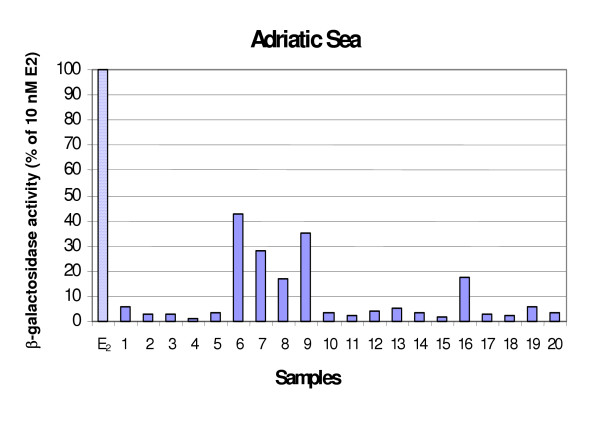
**b-galactosidase activity induced by tissue extracts of seafood from the Adriatic Sea**. Results are expressed as percent activity induced by 10 nM E2.

**Figure 7:**
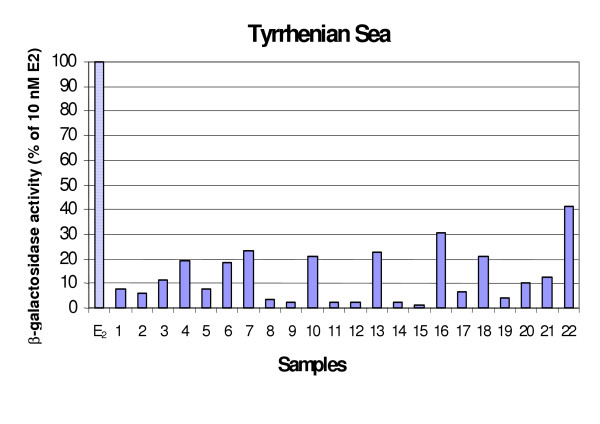
**b-galactosidase activity induced by tissue extracts of seafood from the Tyrrhenian Sea**. Results are expressed as percent activity induced by 10 nM E2.

The b-galactosidase activity elicited by seafood extracts is reported in Figure 6 and Figure 7. Thirty-eight percent of seafood samples showed ER-mediated responses higher than 10% E2. The greatest response measured was 42.96% of the activity elicited by the natural hormone. Tyrrhenian samples were more frequently positive than Adriatic ones (50% versus 25%), although the analysis of variance did not denote any significant differences between the agonistic activity (*p *= 0.13). *S. scombrus*, *O. vulgaris*, *P. phycis*, *M. barbatus *were the more frequently inducing species in Tyrrhenian samples, while for the Adriatic samples the estrogen-like activity was mainly due to *M. merluccius*.

### PCBs and b-galactosidase activity correlation

The content of total PCBs and b-galactosidase activity did not show any significant correlation (a negative borderline correlation was observed, *p *= 0.07).

The PLS analysis showed that in samples with estrogenic activity higher than 10% E2, the most observed combination of these contaminants was PCB 28, PCB 101, PCB 153, PCB 180.

However, total PCBs concentration explained only 33% and 15.77 % of variance, respectively in samples from the Tyrrhenian and the Adriatic Sea.

To better investigate these results, the β-galactosidase activity of individual PCBs was assessed in the yeast assay. The results are showed in Table 2. The b-galactosidase activity of PCB standards ranged from 18.35 % (PCB 101) up to 85.88 % (PCB 118), with PCB 118 resulting the most estrogenic in the yeast assay. This congener belongs to the class of 12 PCBs identified by the WHO as "dioxin-like" because of their toxicity and certain features of their structure which make them similar to 2, 3, 7, 8-tetrachlorodibenzo-p-dioxin (2, 3, 7, 8-TCDD).

**Table 2:** b-galactosidase activity induced by PCB congeners (standard solutions)

**Congeners**	**Concentrations μg/ml**^a^	b**-galactosidase activity (percent of 10 nM E2)**
PCB 28	5.0	31.10 ± 6.58
PCB 52	5.0	31.96 ± 7.82
PCB 101	5.0	18.35 ± 4.26
PCB 118	5.0	85.88 ± 19.79
PCB 138	0.05	20.95 ± 7.82
PCB 153	0.05	26.37 ± 5.63
PCB 180	5.0	22.92 ± 8.67

^a^Congeners 138 and 153 were assayed at 0.05 μg/ml because at higher concentrations they strongly inhibited yeast cells growth

The predicted response according to the individual content of PCBs measured in each seafood sample was calculated. Comparison between predicted and measured enzymatic activity showed a statistically significant difference depending on the two areas. In the Tyrrhenian Sea enzymatic activity measured in the samples was higher than the expected activity, while an inverse correlation was found in the Adriatic Sea. The biological effects of polychlorinated biphenyls (PCBs) are often similar to (although less potent than) those of TCDD by activating the aryl hydrocarbon (Ah) receptor. Additionally, some PCBs or mixtures of PCBs exhibit agonistic activity, whereas others are actually antiestrogenic [2,25,26]. Thus, the estrogenic activity of each congener was determined but the prediction of the effect of the same congener *in vivo *may be extremely difficult, depending on the interactions in a complex environmental mixture.

In this study we analysed the content of PCBs because of their well-documented ability to influence the endocrine system. However, fish tissue may contain a mixture of several environmental compounds other than PCBs interfering with the endocrine system due to the widespread contamination of surface waters with scarcely treated urban and industrial waste that could have additive, synergistic or antagonistic effects. This may account for the final biological activity observed in the samples. Actually, the United States Environmental Protection Agency (US-EPA) estimates there are more than 87,000 of potential EDCs.

Nevertheless, detecting so many chemicals would take an unreasonable investment of time and resources, so it is necessary to develop screening programs using short term bioassays to assess the risk of exposure for biota to endocrine disrupting chemicals through the environment and diet.

Finally, we underline that endogenous hormones could interfere in the estrogen-like activity elicited by animal organic extracts, marine organisms included as recently pointed out by some authors [27]. It is known that in marine organisms, estrogen level may vary depending on differences in species, sex, age, life cycle and season and we tested a raw fat extract in which endogenous hormones are still present. Thirteen species with different habitat and reproductive periods were analysed, and for three species (*M. barbatus*, *P. phycis*, *S. scombrus*) collected both in the Adriatic Sea and in the Tyrrhenian Sea in the same sampling season, statistically significant differences (Fisher's exact test: p < 0.004) in β-galactosidase activity were observed when considered on the whole (Table 3). This result may indicate the role of the aquatic environment in bioaccumulation of xenoestrogens.

**Table 3:** Comparison of the estrogenic activity in the same species from the two habitats

**Species**	**Adriatic Sea**		**Tyrrhenian Sea**		
	Number of samples	Number of positive samples	Number of samples	Number of positive samples	Fisher's exact test
*M. barbatus*	2	-	3	2	p > 0.3
*P. phycis*	5	-	4	2	p > 0.16
*S. scombrus*	4	-	3	2	p > 0.14
Total	11	0	10	6	p < 0.004

## Conclusion

Most studies have been focused on the evaluation of the content of environmental contaminants such as PCBs and related persistent organic pollutants (POPs) into tissues of fish and other aquatic organisms [5,7,23,28-30] or on reproductive effects due to *in vivo *exposure in natural environment [31,32] or to specific sources of pollution [33-35].

In our monitoring we detected a generally low PCBs content in most seafood samples and alone they can not justify the estrogenicity of the extracts. The approach proposed in this work, namely to measure the overall estrogenicity of chemicals each presented at low concentrations, may suggest the probable intake of estrogen-like chemicals for humans.

Then, a useful application of the yeast assay could be aimed to direct chemical analyses to only biologically active samples as a first monitoring level. This bioassay may provide a useful integration to chemical approach, and could be used to identify edible seafood exposed to estrogenic organic chemicals, depending on geographical natural habitat. As yet, however, there is a paucity of analytical data on extracts of edible marine organisms exhibiting estrogenic activity. Fish products may represent an important dietary source of environmental contaminants with endocrine activity to humans, particularly when they represent a relevant part of food intake [11,36]. Many compounds may be present in the environment in trace amounts, but have high biological activity. It is important to assess health risk for biota and the level exposure to environmental contaminants. The foetus and the new-borns in humans are particularly vulnerable to pollutants exposure due to transplacental and lactational transfer of maternal burdens at critical periods of development [37]. The scientific evidence demonstrated a link between chronic exposure to low concentrations of chemicals through the environment or the food-chain and reproductive animal health [31,34,38]. Subtle health effects have been documented in certain Arctic populations exposed to a variety of contaminants present in the food chain (in traditional foods), particularly mercury and PCBs and the greatest concern is for fetal and neonatal development [37,39,40]. The possibility that bio-accumulative properties of persistent organic chemicals with hormone-like activity and the chronic low level exposure may contribute to overall breast cancer risk in women, as well as reproductive and developmental effects in humans [10,41] has heavy implications for the prevention of these diseases in western countries.

## Abbreviations

EDCs: endocrine disrupting chemicals

PCBs: polychlorinated biphenyls

WHO: World Health Organization

ICES: International Council for the Exploration of the Seas

US-EPA: United States Environmental Protection Agency

E2: 17β-estradiol

DMSO: dimethylsulfoxide

PCA: principal component analysis

PLS: partial least square regression

GC-MS: gas chromatography-mass spectrometry

GC-EDC: gas chromatography coupled with electron capture detector

## Competing interests

The author(s) declare that they have no competing interests.

## Authors' contributions

SG and BP carried out the biological analyses and helped to draft the manuscript, TC collected the samples and performed the chemical analyses, MC provided statistical data analysis, RAC participated in the conceiving of the study, DR participated in the design of the study and in coordination and led the writing of the manuscript. All authors read and approved the final manuscript.
